# Experimental data suggest between population reversal in the condition dependence of two sexually selected traits

**DOI:** 10.1038/s41598-025-88720-y

**Published:** 2025-02-04

**Authors:** Gergely Hegyi, Miklós Laczi, Gyula Szabó, Dóra Kötél, Fanni Sarkadi, János Török

**Affiliations:** 1 Behavioral Ecology Group, Department of Systematic Zoology and Ecology, ELTE Eötvös Loránd University, Pázmány Péter sétány 1/C, Budapest, 1117 Hungary; 2 HUN-REN-ELTE-MTM Integrative Ecology Research Group, Pázmány Péter sétány 1/C, Budapest, 1117 Hungary; 3 Barn Owl Foundation, Temesvári út 8, Orosztony, 8744 Hungary; 4 Lendület Ecosystem Services Research Group, Institute of Ecology and Botany, HUN-REN Center for Ecological Research, Alkotmány út 2-4, Vácrátót, 2163 Hungary

**Keywords:** Sexual selection, Social evolution, Behavioural ecology, Evolutionary ecology

## Abstract

When viewing mate choice as a process of adaptive evolution, the condition-dependence of sexual ornaments represents a central pillar. Experimental tests of condition-dependence are few and refer to one population per species. The first brood size manipulation experiment aimed to test ornament condition-dependence had been reported from a Swedish population of collared flycatchers. Here we report a similar experiment conducted in a Hungarian population, examining the change of white plumage patch sizes of male parents by the next year and the patch sizes of male offspring in adulthood. The results consistently indicate that experimentally modified reproductive effort affects male wing, but not forehead patch size. To the contrary, previous results from the Swedish population indicated significant effects on forehead, but not wing patch sizes. Both patches are sexually selected in both populations, so the diverging results offer the first experimental suggestion of a trait by population crossover in the information content of two sexual ornaments. We conclude that explaining why some ornaments are condition-dependent is still far ahead, and further, preferably experimental population comparisons would be helpful.

**Supplementary Information:**

The online version contains supplementary material available at 10.1038/s41598-025-88720-y.

## Introduction

Dependence on physiological or nutritional condition is a well known attribute of sexually selected ornamental traits, a characteristic that arises from their putative production or display costs^1^. Due to the ensuing additive genetic links between sexual ornament expression and aspects of physiological or nutritional state^2,3^, condition-dependence may have evolutionary consequences, contributing to the observed effects of sexual signaling on adaptation and population persistence^4,5^. Condition-dependence has multiple interpretations but it certainly seems widespread^6^. Due to (1) the multiple traits and factors influencing condition and (2) individual differences in overall resource acquisition^7^, as well as (3) possible reversed causality between condition and signal expression^8^, condition-dependence can only be confirmed experimentally. But manipulation of condition is difficult both for dynamic traits due to the transient nature and varying timescale of condition effects in such systems^9^, and for more static traits because such traits are often developed in relatively inaccessible stages of the life cycle^10^ and are subject to degradation thereafter^11^.

The information content of sexual ornaments is strongly determined by the factors limiting their expression and thereby their proximate basis, giving rise to additional complications. Coloration and its information content, for example, may be attributable to pigment deposition^12,13^, a base structure^14,15^, pigment-containing but structurally dominated setups^16,17^ and finally white traits which do not involve pigmentation, only structure^18,19^. Furthermore, the ornamented phenotype, such as the plumage of a male bird, typically involves multiple conspicuous traits that often play different roles^20^, are partly non-functional^21^ or are integrated into composite signals^22^. It is interesting, for example, what happens to trait information content when the relative roles of the same traits differ among populations^23,24^, and experimental data along this line are largely lacking. However, if we examine the condition-dependence of two ornaments, both of which are sexually selected in two different populations, the extent to which proximate determination constrains information content may become especially clear. We are unaware of any such comparison in the literature.

Studies of within-individual flexibility and proximate determination of ornamentation typically focus on bird coloration. Experimental studies of environmental sensitivity may employ trait-specific, often physiological methods^25,26^, but the perhaps most easy-to-generalize approach is to manipulate nutritional condition in a standard way. Keeping and molting individuals in the laboratory is feasible in only a limited range of species^27,28^, so nutritional manipulation is often done by brood size alterations. The easiest trait to measure then is offspring coloration, but only a few offspring traits quantified in such studies were retained to adulthood and could act as sexual signals^29–32^. A more difficult, but often more relevant object to measure is change in the ornamentation of parents by the next year, and we are aware of only four such experiments so far (collared flycatchers *Ficedula albicollis*^33,34^, house sparrows *Passer domesticus*^35^, eastern bluebirds *Sialia sialis*^36,37^, pied flycatchers *Ficedula hypoleuca*^38^; see also ref. 39 for a brood removal experiment in blue tits *Cyanistes caeruleus*). Finally, the most difficult thing to measure is the adult ornamentation of nestlings returning from the manipulated broods, with only two examples known to us so far (collared flycatchers^33^, barn swallows *Hirundo rustica*^40^; see also ref. 41 for a food quality manipulation experiment and ref. 42 for experimental data on a non-ornamental color trait).

Here we present data on two ornamental traits, the white forehead and wing patch sizes of males from a brood size manipulation experiment conducted in five breeding seasons in a Hungarian collared flycatcher population. Brood sizes in the main breeding period are typically five to seven. We increased and reduced them by two nestlings, kept comparable, non-manipulated controls, and measured at the focal nests the patch sizes of both the parents in the next year and their recruiting offspring in their adulthood. However, the unique feature of our study is a possibility for a population comparison (Gotland, Sweden versus Pilis-Visegrádi Mountains, Hungary). Previous studies in both populations showed that social mate acquisition is significantly associated with both forehead and wing patch size^34,43^. A brood size manipulation experiment in Gotland yielded the first evidence of ornament-related reproductive costs, thereby demonstrating that forehead patch size is condition-dependent^33^, but the same experiment had no effect on wing patch size^34^. Paradoxically, in the Hungarian population, correlative studies suggested that wing, but not forehead patch size is associated with nutritional condition, and that the two traits are also inversely related to male viability^44–46^. Here we present a comparable brood size manipulation study done in the Hungarian population. Therefore, the results presented below offer the first possibility to our knowledge to compare, based on experimental data, the condition dependence of two sexually selected ornamental traits between two populations of the same species, i.e. to examine the possibility of a trait by population crossover in ornament information content.

## Results

**Fig. 1:**
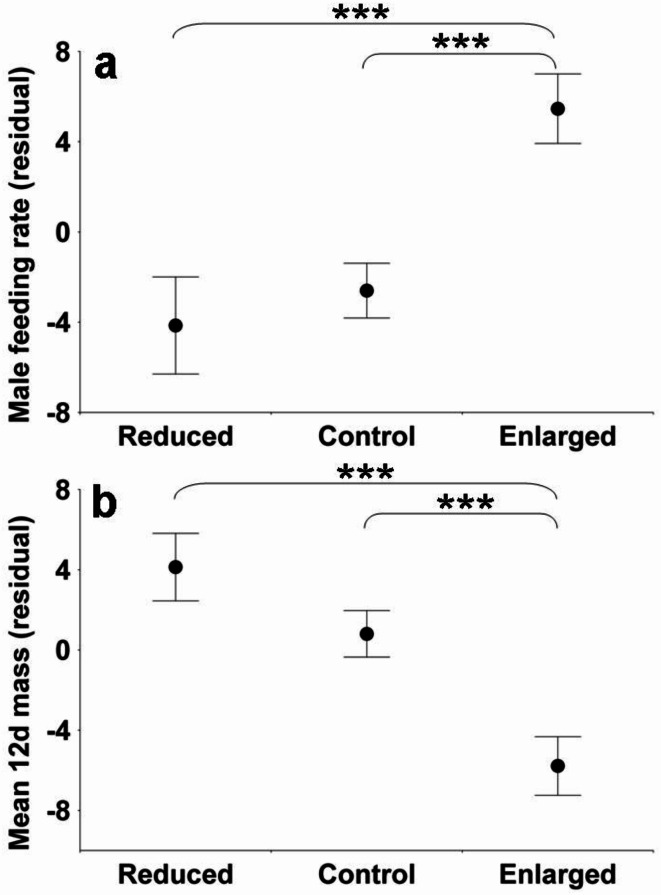
Possible mediators of intra- and inter-generational costs of reproduction: hourly male feeding rate (number of visits; 13, 17 and 18 data points for reduced, control and enlarged groups) of ten day old nestlings (**a**) and mean twelve day nestling mass (0.1 g; 40, 39 and 34 data points respectively) (**b**) in relation to brood size manipulation; means ± standard error. Residuals are shown from a linear model with year as a factor. Asterisks indicate the level of significance for the pairwise comparison spanned by the respective bracket (***, *p* < 0.001).

We ran general linear models which always contained year and manipulation category as factors, sometimes with other correction variables. Detailed model structures and results are shown in Table 1. We omitted yearling birds in subadult plumage and focused on older birds, hereafter called “adults”. Forehead or wing patch size of males in the year of manipulation was unrelated to either manipulation category or year. Hourly male feeding rate strongly reacted to manipulation (Fig. 1a), with a significant year effect. Linear models for pairs of manipulation categories indicated that males with enlarged broods performed larger numbers of feedings than those of the other categories (control vs. enlarged *p* = 0.001; reduced vs. enlarged *p* < 0.001), while the reduced-control difference was non-significant (*p* = 0.234). A similar result but in the opposite direction was obtained for mean 12 day nestling masses, with effects of both manipulation (Fig. 1b) and year, and two pairwise comparisons significant (reduced > enlarged *p* < 0.001; control > enlarged *p* < 0.001), but the third one not (reduced vs. control *p* = 0.118).

**Table 1:** Effects of our experiment on parental feeding rate, nestling mass, and the patch sizes of male parents and recruits.

	Year					Manipulation					Original patch size					Feeding rate				
	F	df	ES	CI low	CI up	F	df	ES	CI low	CI up	F	df	ES	CI low	CI up	F	df	ES	CI low	CI up
Original FPS	2.06	4, 43	0.401	0.132	0.615	0.32	2, 45	0.119	-0.171	0.390	NA	NA	NA	NA	NA	NA	NA	NA	NA	NA
Original WPS	0.71	4, 43	0.249	-0.038	0.498	1.79	2, 45	0.271	-0.014	0.516	NA	NA	NA	NA	NA	NA	NA	NA	NA	NA
Feeding rate	2.75*	4, 38	0.474	0.219	0.668	12.15***	2, 38	0.625	0.414	0.772	NA	NA	NA	NA	NA	NA	NA	NA	NA	NA
Mean 12d nestling mass	4.62**	2, 106	0.385	0.216	0.532	11.23***	2, 106	0.418	0.253	0.560	NA	NA	NA	NA	NA	NA	NA	NA	NA	NA
DFPS (with manipulation)	0.28	4, 43	0.159	-0.131	0.424	1.11	2, 45	0.217	-0.071	0.472	1.76	1, 46	-0.192	-0.451	0.098	NA	NA	NA	NA	NA
DWPS (with manipulation)	1.05	4, 40	0.309	0.027	0.545	5.55**	2, 44	0.449	0.189	0.650	5.85*	1, 44	-0.343	-0.571	-0.065	NA	NA	NA	NA	NA
DFPS (with feeding rate)	0.35	4, 41	0.185	-0.105	0.446	NA	NA	NA	NA	NA	1.43	1, 43	-0.180	-0.441	0.110	1.19	1, 43	-0.164	-0.428	0.126
DWPS (with feeding rate)	0.76	4, 40	0.265	-0.021	0.511	NA	NA	NA	NA	NA	3.12	1, 42	-0.263	-0.509	0.023	8.36**	1, 43	-0.403	-0.617	-0.135
Recruit FPS	1.21	4, 10	0.572	0.084	0.839	1.66	2, 12	0.465	-0.062	0.789	NA	NA	NA	NA	NA	NA	NA	NA	NA	NA
Recruit WPS	0.43	4, 8	0.419	-0.119	0.767	5.44*	2, 12	0.690	0.274	0.888	NA	NA	NA	NA	NA	NA	NA	NA	NA	NA
Relative recruit FPS	2.53	4, 9	0.728	0.321	0.908	0.35	2, 11	0.246	-0.327	0.687	NA	NA	NA	NA	NA	NA	NA	NA	NA	NA
Relative recruit WPS	0.23	4, 7	0.343	-0.230	0.739	10.81**	2, 11	0.814	0.499	0.939	NA	NA	NA	NA	NA	NA	NA	NA	NA	NA

FPS, forehead patch size; WPS, wing patch size; D, change; ES, effect size; CI, 95% confidence interval; *, *p* < 0.05; **, *p* < 0.01; ***, *p* < 0.001.

**Fig. 2:**
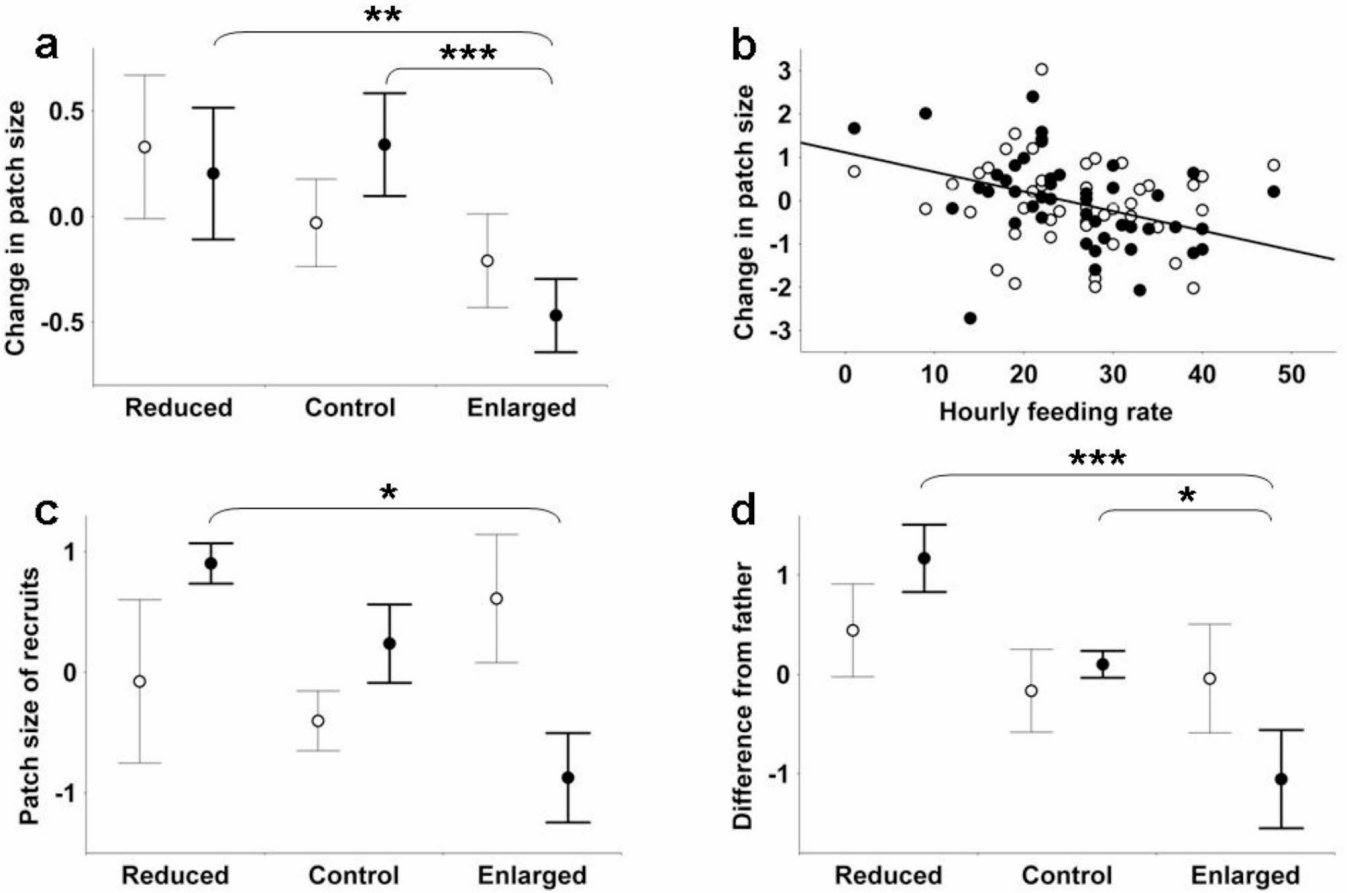
Dynamics of male white patch sizes in a brood size manipulation experiment. Change of parental patch size in relation to manipulation (**a**) and feeding rate (**b**), and the effects of manipulation on the patch sizes of recruits (**c**) and on their deviation from paternal patch size (**d**). Open circles refer to forehead patch size and filled circles to wing patch size. The box plots show means with their standard errors. The number of data for reduced, control and enlarged groups is 13, 17 and 18 for patch size change and 3, 7 and 5 for recruit ornamentation. The scatterplot shows the least squares linear regression line for wing patch size only, because there was no significant relationship for forehead patch size. For the sake of comparability, patch sizes or deviation values were standardized within the given sample to a mean of zero and a standard deviation of one. Therefore, the unit of the Y axis is always standard deviation. Asterisks indicate the level of significance for the pairwise comparison spanned by the respective bracket (*, *p* < 0.05; **, *p* < 0.01; ***, *p* < 0.001).

When controlling for year, change in forehead patch size in the male parent showed no significant treatment effect (Fig. 2a open circles). Change in wing patch size was significantly negatively related to original patch size and also manipulation (Fig. 2a filled circles). The reduced-control comparison was non-significant (pairwise GLM, *p* = 0.949), while males rearing enlarged broods grew smaller wing patches relative to their original value than those rearing reduced (*p* = 0.012) or control broods (*p* = 0.005). Male wing patch size change was also negatively related to hourly nestling feeding rate observed during the experiment (Fig. 2b filled circles and regression line), while there was no such relationship for forehead patch size (Fig. 2b open circles). Feeding rate is related to manipulation and therefore the results for the two variables are not statistically independent. We therefore note that the effects of both manipulation and feeding rate on wing patch size change remain significant if we reduce the critical p value to 0.025.

The adult recruits exhibited no effect of the year of birth on any patch (Table 1). There was a significant treatment effect on wing patch size (Fig. 2c filled circles) with no significant reduced-control difference (Scheffé tests, *p* = 0.490) but significant reduced > enlarged (*p* = 0.029) and marginal control > enlarged differences (*p* = 0.091). There was no significant pattern for forehead patch size (Fig. 2c open circles). When looking at difference from the father’s patch size, the manipulation effect became stronger for wing patch size (Fig. 2d filled circles). The reduced > control difference was marginal (*p* = 0.091) while the control > enlarged and reduced > enlarged differences were significant (*p* = 0.043 and *p* = 0.003 respectively). The manipulation effect remained non-significant for forehead patch size (Fig. 2d open circles). Father’s patch size was non-significant when entered into any of these models (details not shown). Finally, recruit patch sizes at two years of age or later did not show the expected positive relationship with body mass at eight or twelve days of age (*n* = 15; forehead patch and eight day mass *r*=-0.39, *p* = 0.151, forehead patch and twelve day mass *r*=-0.53, *p* = 0.041; wing patch and eight day mass *r* = 0.16, *p* = 0.572, wing patch and twelve day mass *r* = 0.17, *p* = 0.539).

## Discussion

In our collared flycatcher population, both intra- and inter-generational manipulation effects were significant for wing patch size but non-significant for forehead patch size. There was a particularly consistent effect of brood size enlargement on both parents and recruits. Moreover, the experimental effect on recruit wing patch size became clearer when paternal patch size was controlled for. These results confirm previous correlative suggestions that forehead patch size has much lower plasticity and a weaker link to nutritional condition than wing patch size^43,44,46,47^. It might be worth highlighting the apparently different mechanisms of condition-dependence observed in brood-rearing males versus recruit males. In parents, relative wing patch size declined in proportion to feeding effort, even though the reduced group did not differ from controls in either feeding rate or patch size change. Therefore, the intra-generational experimental effects can truly be considered as costs of reproduction to males in terms of condition-dependent ornamentation, as originally suggested^33,48^. Considering recruits, growing up in manipulated broods had even more clear-cut effects on their adult wing patches than for their male parent. However, this effect is apparently independent of direct influences through mass growth. The absence of a direct effect is logical because the wing patches we measured on the adult recruits were grown at least a year after their fledging. The alternative mechanism causing the recruit ornamentation difference needs further study. We suggest that it could be related to long-lasting developmental stress effects that have been shown to have short-term ornamentation consequences^49^, but also lifetime organizational effects on reproductive investment^50^ in other species.

To the best of our knowledge, our results provide the first tentative comparison of condition-dependence in a sexually selected ornamental trait pair between two populations based on experimental data. In both the Swedish population of Gotland and the Hungarian population of the Pilis-Visegrádi Mountains, forehead and wing patch sizes of male collared flycatchers are similarly positively selected in terms of social mate acquisition^34,43^. In Gotland, male forehead patch size strongly reacted to manipulation of the size of the reared brood, and there was a similar but weaker effect for recruit patch sizes, demonstrating for the first time intra- and intergenerational costs of reproduction in terms of a sexually selected trait^33^. Wing patch size, in contrast, showed no effect of the manipulation, as described in ref. 34 (only intra-generational data were shown). Results for condition-dependence from our population indicate condition-dependence for wing but not forehead patch size, and they therefore suggest a between-population crossover in the plasticity of two similarly sexually selected traits.

We must make three cautionary notes concerning these conclusions. First, the two experiments (Sweden versus Hungary) were made three decades apart and trait information content may have evolved during this period, thereby invalidating the comparison. Indeed, a rapid change in the information content of yearling (but not adult) forehead patch size was described earlier in our birds^45^. However, there was a previous experiment in our population^51^ roughly coincident with the Swedish study^33^, where only forehead patch sizes were measured yet. Data from this experiment are in line with the present ones, showing no trace of experimental effect (see the OSM for details). In addition, our current results for wing patch size are in agreement with correlative data collected two decades earlier^46^, both of them suggesting significant condition-dependence. Therefore, rapid evolution may not have confounded the observed population difference for either trait. Second, a direct statistical comparison of the manipulation responses would be the most convincing demonstration of the population difference, and this is not possible because we could not obtain the Swedish experimental data analyzed three decades ago. However, all manipulation effects for both traits in both populations (plus our previous correlative analyses) confirm the population difference, and the likelihood of such results arising by chance is very low. A third concern is that the earlier brood size manipulation experiment we did in our population yielded strongly divergent effects on life history traits in the years of caterpillar gradation^51^. This year quality effect apparently prevented the life history optimization seen in the Swedish data (ref. 52 versus ref. 51). However, there was no gradation year during our present experiment, so this effect does not confound the population comparisons. Also note that year effects on original patch sizes, patch size changes and recruit patch sizes were all non-significant in this data set.

Another possible critique refers to the similar size and similar (depigmented) nature of forehead and wing patches. Is it possible that the two patch sizes are part of the same “overall patch size” stimulus to females? If so, trait-specific information content, even if present, would not be evolutionarily important. Correlation of both traits with pairing patterns in both populations regardless of information content could just reflect this “pooled” functioning and then it would not require any elaborate explanation, including experimental population comparisons such as our work. We argue that the probability of such a scenario is very low in this species, as judged from background knowledge and our own previous findings.

First, the forehead patch is large and mostly independent of age in males but it is vestigial or absent in most females. Wing patch size, in contrast, is measurable in both sexes, but it is twice as large in adult males than in yearling males or females. Such differences in age- and sex-dependence can be expected to pose difficulties in the context of “pooled” evaluation. Second, and more importantly, the two patches are molted in different seasons (forehead in winter, wing in summer^53^) and only the wing patch is present in the non-breeding plumage of males, thereby also continually “reminding” females that wing and forehead patches are different traits. Third, the two patches are related to the macroclimatic variables of their respective (different) season of molt^47^. There is also a drastic difference in their among-year variation, with seemingly random patterns in wing patch size but strong wave-like fluctuation in forehead patch size (G. Hegyi, M. Herényi, M. Laczi, G. Markó, G. Nagy, B. Rosivall, E. Szöllősi and J. Török, unpublished data). Therefore, females are confronted with very different mean relative sizes of the two patches in different years. Finally, and most importantly, some patterns of sexual selection seem to differentiate between the two patches. On the one hand, there is correlative evidence that females focus on the patch that is larger on average at the population level in the given year, and this requires separate “search images” for the two patch sizes^47^. On the other hand, intrasexual competition experiments with dummy birds indicated that wing patch size is an important determinant of responses by the resident in both males^54^ and females^55^, while male forehead patch size is unimportant in the context of territorial responses^54^. In sum, the two patches do seem to represent different signal traits, thereby validating the assessment of concordance between their relative information content and functioning.

Among the four previously conducted experiments examining intra-generational costs of reproduction on ornamentation, three showed a negative tendency of ornamentation changes to the next year with increasing brood size manipulation (collared flycatcher forehead patch size^33^, house sparrow bib size^35^, eastern bluebird blue brightness^36^), while one study obtained an opposite tendency, and even that only for yearling males (pied flycatcher plumage darkness^38^). There was also supportive evidence from a brood removal experiment in blue tits^39^. Concerning inter-generational costs, forehead patch size of Swedish collared flycatcher recruits reacted to the manipulation of their natal brood size in the appropriate direction^33^, while tail length of barn swallow recruits was unaffected by the same type of manipulation^40^. In addition, among nestling color traits presumably retained until the first breeding round, brood size manipulation affected at least one measured trait in three of four species (blue tit^30^, eastern bluebird^31^, northern flicker *Colaptes auratus*^32^; but not in the barn owl *Tyto alba*^29^). Therefore, both intra- and inter-generational costs of reproduction on male ornamentation seem clear-cut, but note that many species with assumedly condition-dependent ornamentation have not been subjected to such manipulations.

Another interesting pattern is that most studies with positive results also measured at least one ornamental trait that was unaffected by the manipulations, introducing one more layer of complexity. The literature contains brood size manipulation data and therefore comparable experimental evidence for the condition-dependence of multiple ornamental traits from very few species, as listed above. Population differences within species form another layer of complexity. We are aware of two species in which population difference in the functioning of the same ornamental traits has been suggested. In common yellowthroats *Geothlypis trichas*, female choice was predicted by black mask size in one population and plumage yellowness in another population, while the outcome of male competition was determined by mask size in both populations, and the two populations were phenotypically similar^56^. Information content was examined only correlatively, but both physiological^57^ and genomic data^24^ strongly suggested that individual quality is more robustly indicated by the trait that is subject to mate choice in the given population. In barn swallows, the known sexually selected trait (and here also the dominant visible ornament) is tail length in northern European birds^58,59^ but ventral plumage redness in North America^60,61^. Population-specific data on the information content of these traits are correlative and are less consistent than the sexual selection data^62,63^. But in these examples, information content is not particularly enlightening given that only one trait is sexually selected in either population, and we do not expect the same level of information content for traits not under sexual selection.

Based on our results, it seems that, out of two similarly sexually selected traits of very similar origin (white plumage patches), condition-dependence applies to one trait in one population and the other trait in the other population. This finding does not agree with previously found robust relationships between trait information content and sexual selection function within populations^43,64^, across species^6,13^, and across populations within species^24,57^. There may be confounding factors on each level, such as trait integration when working within populations^22^, evolutionary constraints when comparing species^65^ and the presence of non-functional traits when comparing populations^63^, but our results nevertheless call for further research.

The currently emerging main line of thinking with respect to multiple ornamental traits of the same species is to find the level of variation and covariation that is relevant to sexual selection^22,66^. We suggest that the individual trait approach may still yield much new insight, especially for trait pairs, such as forehead and wing patch size in our study species, that vary differently and are not functionally integrated either^47^. Studying trait condition dependence (and not only function) in multiple populations of widely studied species with respect to sexual selection^67,68^ may show us the relative explanatory power of trait information content as a determinant of sexual selection, when compared to other potential determinants like environmental constraints (phenology^69^, habitat attributes^70^), evolutionary constraints such as population interrelatedness and dispersion^71,72^ or cognitive differences^73,74^. Such population comparisons may explore a hitherto largely unknown layer of variation in sexual selection as a putatively adaptive evolutionary process^24,75^.

## Methods

### Ethics statement

We conducted this study with ethical approval by the institutional animal welfare committee of Eötvös Loránd University (permit numbers T–012/2015, T-020/2017) and with research permit from the regional nature conservation authority (permit numbers KTVF 509-4/2012, PE-06/KTF/920-7/2018). We conform to relevant national guidelines and regulations. The reporting of our work conforms to the ARRIVE guidelines.

### Field methods

The experiment was performed in our long-term study plots of nest boxes in the Pilis-Visegrádi Mountains in the years of 2015, 2016, 2019, 2021 and 2022. Manipulation was done by an asymmetric exchange of nestlings at two days of age between two collared flycatcher broods of the same hatching date and clutch size and a brood size difference of a maximum of one nestling. We moved two nestlings from the enlarged to the reduced brood and four nestlings in the reverse direction while individually marking all nestlings by removing tufts of down from the head or shoulders. The resulting broods contained similar proportions of own and foreign young. A third, non-manipulated brood (see previous findings in ref. 51 for justification) that had hatched at the same time and complied with the same size criteria as above was used as a control brood in which nestlings were also individually marked at two days of age. Identification and ringing were done at eight days of age.

To assess the effect of experiment on the nestlings, these were individually weighed at two, eight and twelve days of age. Feeding rates, a measure of parental reaction to the manipulation, were quantified from video recordings of one hour of activity at ten days of nestling age (see details in ref. 76). Parents were caught and measured shortly after making these video recordings. Maximum height and width of the forehead patch size of males, and visible lengths of white on the outer vanes of primaries 4 to 8 on the right wing were measured by caliper to the nearest 0.1 mm (for validation and repeatability, see refs. 44 and 46). Wing patch size was estimated as the sum of the measured segments (a length measure, in mm) and forehead patch size as maximum height multiplied by maximum width (an area measure, in mm^2^). Returning young were measured in the same way during the yearly captures of the breeding population. Wing patch size is half as large in yearlings as in older birds^46^. More importantly, the forehead patch size of yearlings is negatively related to the father’s patch size, so it is very difficult to interpret^45^. Therefore, we were obliged to restrict the analyses of recruits to two-year-old or older birds. This makes our analyses conservative by only detecting the long-term effects of postnatal conditions.

Over the five years, we created 44 experimental trios (7 to 10 per year), with a total of 132 broods. Due to some complete brood failures, nestling masses could be analyzed and parents captured in 113 broods (40 reduced, 39 control and 34 enlarged). Due to the approximately 50% return rate for adults and less than 10% recruitment rate of nestlings^51^, accompanied by the presence of yearlings and missing measurements due to e.g. ongoing molt, the number of males we could use for analyzing parental patch size change in relation to manipulation and feeding rate was 48 (13 reduced, 17 control and 18 enlarged). For adult recruit ornamentation, the same number was 15 (3 reduced, 7 control and 5 enlarged). There was no pseudoreplication in the recruit data set as all analyzed recruits originated from different fathers and broods.

### Statistical analyses

Statistical analyses were done in Statistica 5.5 (StatSoft, Inc.). Feeding rates of male parents and their patch sizes in the year of manipulation were analyzed with year and manipulation as factors. Mean 12 day nestling masses were analyzed for all broods in which nestlings survived to fledging, using the same model structure. Changes in parental patch size were analyzed similarly but also including original patch size as a covariate. The patch sizes of adult recruits were analyzed with year and manipulation as factors. To reduce the probability of chance patterns, the effect of manipulation on recruit patch size was also tested by using the differences of recruit patch size from the patch size of the male parent at the brood of origin as a dependent variable. In the case of yearling fathers (non-interpretable patch sizes^45^), we used a patch size value from their following breeding year where available. This issue affects one control and one enlarged brood, and the single next-year manipulated father ornament value (that could itself be affected by the manipulation) makes this analysis conservative. The conclusions are the same without these data. Interactions between year and manipulation could not be included because not all manipulation groups produced returning adults/recruits in all years. This is unlikely to cause interpretational problems considering that there was no caterpillar gradation in the experimental years and neither patch sizes nor their changes showed any significant year effect in any of the analyses of this data set (see Table 1 below). General linear models involved stepwise removal and reintroduction of the non-significant terms based on their significance. We also correlated recruit patch sizes with their eight day mass (at peak feather growth) and twelve day mass (value before fledging) using Pearson correlations.

## Electronic supplementary material

Below is the link to the electronic supplementary material.

Supplementary Material 1

Supplementary Material 2

Supplementary Material 3

## Data Availability

Data are provided in the supplementary information files.
