# Supplementary material for: Experimental data suggest between population reversal in the condition dependence of two sexually selected traits
Source: Sci Rep. 2025 Feb 4;15:4264. doi: 10.1038/s41598-025-88720-y (PMC11794571; doi:10.1038/s41598-025-88720-y)
Supplement: Supplementary file 1 — Supplementary Material 1 [file 41598_2025_88720_MOESM1_ESM.doc]

Online Supplementary Material to

Experimental data suggest between-population reversal in the condition-dependence of two sexually selected traits

Gergely Hegyi, Miklós Laczi, Gyula Szabó, Dóra Kötél, Fanni Sarkadi, János Török

Effects of an earlier brood size manipulation on forehead patch sizes

Methods

The earlier experiment was done in six years from 1988 to 1993, and there were four major differences from the more recent experiment. The first difference was the experimental protocol. The nestlings were moved in an unbalanced manner (only from reduced to enlarged broods), the range of manipulation was broader (reduction and increase by two or four nestlings), and nestlings were not individually marked at the time of manipulation, so the original brood of the recruits could not be confirmed for the enlarged broods. Second, control broods were not associated with the nestling swapped brood pairs, so the analyses had to capitalize on the comparable non-manipulated broods remaining after the manipulations. Third, only the forehead patch size of males was measured at that time, and even that only from 1990. The fourth difference was the presence of two caterpillar gradation years (1992 and 1993) with one magnitude greater caterpillar availability than in average years.

We adjusted the analyses to these conditions as follows. First, we analyzed the full range of manipulation (-4 to +4) and for comparability, also a reduced data set conforming to the more recent experiment (-2 to +2). Second, as control broods, we used all broods with similar clutch size to the manipulated broods that were laid within the range of laying dates observed among the manipulated broods in the given year. Third, due to the time-restricted availability of forehead patch sizes, we used data from 1988 to 1993 for the adult recruits but only from 1990 to 1993 for the male parents. Fourth, we used year type (average or peak food year) as a factor in the analyses instead of raw year. Finally, we did not calculate results for recruit patch size relative to the father due to the lack of individual marking and therefore origin data in the enlarged broods.

Results

The results are shown in Supplementary Table 1. Neither year type nor manipulation was associated with the current forehead patch sizes of males. Forehead patch size change was significantly or marginally related to original patch size, but no other term was significant. Finally, recruit forehead patch sizes showed no significant pattern with manipulation of their brood of rearing. The year type effect on the recruits was close to significance, but it showed an unexpected direction, with a tendency for larger patch sizes for recruits returning from average years than for those returning from peak food years. Therefore, none of the results indicates condition-dependence in forehead patch size, in line with our more recent experimental data.

Supplementary Table 1. Relationships of original forehead patch size, change in patch size, and recruit patch size with year type and brood size manipulation category. General linear models with backward simplification and reintroduction.

|  | Year type | |  |  |  | Manipulation | |  |  |  | Original patch size | | |  |  |
| --- | --- | --- | --- | --- | --- | --- | --- | --- | --- | --- | --- | --- | --- | --- | --- |
|  | F | df | ES | CI low | CI up | F | df | ES | CI low | CI up | F | df | ES | CI low | CI up |
| Original FPS full range | 0.49 | 1, 74 | 0.081 | -0.147 | 0.301 | 0.21 | 4, 71 | 0.108 | -0.120 | 0.326 | NA | NA | NA | NA | NA |
| Original FPS reduced | 0.38 | 1, 51 | 0.086 | -0.189 | 0.348 | 0.28 | 2, 50 | 0.105 | -0.170 | 0.365 | NA | NA | NA | NA | NA |
| FPS change full range | 0.72 | 1, 73 | 0.099 | -0.129 | 0.317 | 0.20 | 4, 70 | 0.106 | -0.122 | 0.324 | 7.28** | 1, 74 | -0.299 | -0.491 | -0.079 |
| FPS change reduced | 0.35 | 1, 51 | 0.083 | -0.192 | 0.346 | 0.21 | 2, 50 | 0.091 | -0.184 | 0.353 | 3.64 | 1, 51 | -0.258 | -0.494 | 0.013 |
| Recruit FPS full range | 3.91 | 1, 53 | 0.262 | -0.004 | 0.493 | 0.24 | 4, 50 | 0.137 | -0.133 | 0.388 | NA | NA | NA | NA | NA |
| Recruit FPS reduced | 2.97 | 1, 47 | 0.244 | -0.040 | 0.492 | 0.44 | 2, 46 | 0.137 | -0.150 | 0.403 | NA | NA | NA | NA | NA |

CI, 95% confidence interval; ES, effect size; FPS, forehead patch size; **, p<0.01

Supplementary Table 2 (separate file). The analyzed data set for returning adult males that were subjected to the experiment as parents

FPS, forehead patch size in sqmm; WPS, wing patch size in mm; Feeding rate, number of feedings to endothermic nestlings per hour

Supplementary Table 3 (separate file). The analyzed data set for returning adult males that were subjected to the experiment as nestlings. One father had no adult ornament data

FPS, forehead patch size in sqmm; WPS, wing patch size in mm; 8d mass, body mass of the nestling at 8 days of age; 12d mass, body mass of the nestling at 12 days of age
